# Hot-starting software containers for STAR aligner

**DOI:** 10.1093/gigascience/giy092

**Published:** 2018-07-31

**Authors:** Pai Zhang, Ling-Hong Hung, Wes Lloyd, Ka Yee Yeung

**Affiliations:** School of Engineering and Technology, Campus Box 358426, 1900 Commerce Street, University of Washington, Tacoma, Washington 98402-3100, USA

**Keywords:** software container, reproducibility of research, cloud computing

## Abstract

**Background:**

Using software containers has become standard practice to reproducibly deploy and execute biomedical workflows on the cloud. However, some applications that contain time-consuming initialization steps will produce unnecessary costs for repeated executions.

**Findings:**

We demonstrate that hot-starting from containers that have been frozen after the application has already begun execution can speed up bioinformatics workflows by avoiding repetitive initialization steps. We use an open-source tool called Checkpoint and Restore in Userspace (CRIU) to save the state of the containers as a collection of checkpoint files on disk after it has read in the indices. The resulting checkpoint files are migrated to the host, and CRIU is used to regenerate the containers in that ready-to-run hot-start state. As a proof-of-concept example, we create a hot-start container for the spliced transcripts alignment to a reference (STAR) aligner and deploy this container to align RNA sequencing data. We compare the performance of the alignment step with and without checkpoints on cloud platforms using local and network disks.

**Conclusions:**

We demonstrate that hot-starting Docker containers from snapshots taken after repetitive initialization steps are completed significantly speeds up the execution of the STAR aligner on all experimental platforms, including Amazon Web Services, Microsoft Azure, and local virtual machines. Our method can be potentially employed in other bioinformatics applications in which a checkpoint can be inserted after a repetitive initialization phase.

## Findings

### Background

With the availability of high-throughput next-generation sequencing technologies and the subsequent explosion of big biomedical data, the processing of big biomedical data has become a major challenge. Cloud computing plays an important role in addressing this challenge by offering massive scalable computing and storage, data sharing, and on-demand access to resources and applications [1, 2]. The National Institutes of Health is launching a Data Commons Pilot Phase to provide access and storage of biomedical data and bioinformatics tools on the cloud [3]. Additionally, software containers have become increasingly popular for deploying bioinformatics workflows on the cloud. Docker [4], an open-source project, has become the *de facto* standard for container software. Docker packages executables with all the necessary software dependencies, ensuring that the same software environment is replicated regardless of the host hardware and operating system. Other container technologies such as Singularity containers have also been proposed to enhance mobility and reproducibility of computational science [5, 6]. Thus, containerization enhances the reproducibility of bioinformatics workflows [7–9]. In the context of cloud computing, the utility of containers comes from the ease with which a virtual cloud cluster can be rapidly provisioned with all of the necessary dependencies for a complicated workflow by simply downloading a set of containers, each of which takes a few seconds to spin up. Recently, Vivian et al. processed more than 20,000 RNA sequencing (RNA-seq) samples from the Cancer Genome Atlas using Docker containers on the cloud [10]. Tatlow et al. used software containers to study the performance and cost profiles of different cloud-based configurations in processing RNA-seq data from public cancer compendia [11].

When containers are deployed, applications are launched *de novo* each time the container is spun up. This means that any initial preparatory steps are repeated each time the container is used. For tasks such as the alignment of reads, these initial steps can be quite substantive as large sets of indices need to be read before alignments can begin. In an automated large-scale deployment, these steps are replicated many times. It would be far more efficient if one could “*checkpoint*” and save containers in states where the application has already completed the initialization steps so as to avoid unnecessary repetitions. One could then “hot-start” workflows from these checkpoints. This is analogous to hot-start polymerase chain reaction (PCR) where all the necessary reagents are premixed awaiting only the addition of the template.

### Our approach

Our key idea is to save and restore memory states in software containers using the Checkpoint Restore in Userspace (CRIU) tool. CRIU freezes a running container and saves the checkpoint as a collection of files on disk [12]. These files can subsequently be used to restore and resume the application from that checkpoint. CRIU was originally developed for Linux but has recently become available for Docker [13]. While it is possible to stop Docker containers with native Docker commands, this process does not preserve the memory state. Although restarting from a ready-to-go state is an intuitive application of checkpointing, we have been unable to find any previous description of using checkpointing as a general method for improving the efficiency of container deployments.

We demonstrate that hot-starting from a saved container checkpoint can significantly reduce the execution time using the spliced transcripts alignment to a reference (STAR) aligner [14, 15] for RNA-seq data analyses. We chose STAR as a proof-of-concept example because it has such a slow initialization step that it includes an option to retain indices in memory for use when aligning many different files. However, our idea of using checkpoints has broad applications in optimizing performance using software containers on the cloud when performing any bioinformatics task where a pause could be inserted to capture a re-usable state.

**Figure 1:**
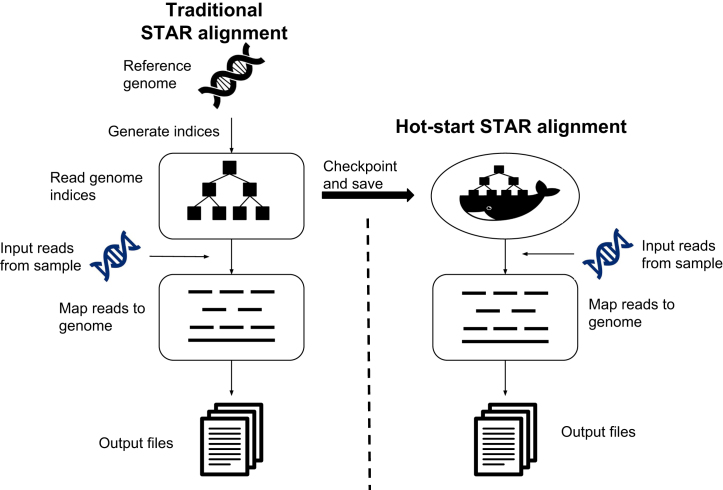
An overview of our approach with and without checkpoints. The left panel shows the two steps of the STAR aligner [14, 15] after the generation of indices. The right panel shows our approach using the CRIU tool that freezes a running container and saves the checkpoint as a collection of files on disk after the genome indices are generated using the reference genome. Our “hot-start” containers use these saved files to restore the application and map the reads from the experimental sample data to the reference.

The STAR aligner consists of several steps. Indices are generated from the reference genome. This is typically done just once using the latest version of the reference. The indices are read in; then, read sequences from a specific experiment sample are mapped to the reference genome. For STAR, reading in the indices is a slow process, and STAR has an option of keeping the indices in memory after they have been generated so that subsequent sequence alignments do not have to repeat the step of reading the indices. We used the CRIU tool to create checkpoints after the indices have been read. Instead of launching a new container and starting STAR from scratch, we restore the container state using CRIU and resume running STAR after it has loaded the indices. Figure 1 shows an overview of our approach with and without using checkpoints.

### Testing

**Figure 2:**
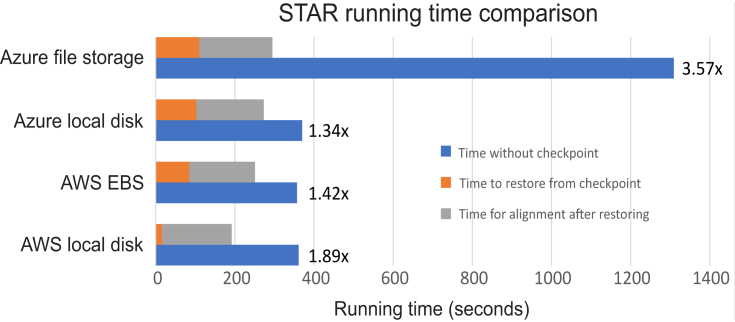
STAR alignment running time comparison with and without checkpoint. The running time is averaged over five runs. We performed our empirical experiments on two cloud platforms: AWS and Microsoft Azure. Both the Azure File Storage and the Amazon EBS represent network disks. We observe that our “hot-start” containers (orange and gray bars) provide a major reduction in execution time, especially on local disks.

To test the checkpointing methodology, we used RNA-seq data generated by Himes et al. These data measure the gene expression changes in human airway smooth muscle cells in response to asthma medications [16]. We compared the time required to align the sequences with a normal container where STAR starts from scratch and the time required when hot-starting from a container checkpoint where STAR has already generated indices. We performed empirical studies on multiple cloud platforms including Amazon Web Services (AWS) and Microsoft Azure, using both local and networked disks. On AWS, we compared performance with data stored on the local host vs Amazon Elastic Block Store (EBS). On Microsoft Azure, we compared the performance with data stored on the local host vs Azure File Storage. Please refer to the Online Methods for details of our experimental setup. Our empirical results are shown in Fig. 2.

Figure 2 shows that the STAR aligner with checkpointing reduces the execution time compared to STAR without checkpointing. The average running times over five separate runs are shown. The raw data, average running time, and standard deviation across the five runs are available as Additional File 2. On AWS, we observed a 1.89x speedup with data stored on the local disk and 1.42x speedup with data on a network disk (Amazon EBS). On Microsoft Azure, we achieved a 1.34x speedup with data stored on the local disk and 3.57x speedup with data on Azure File Storage. With respect to execution time, we show that hot-starting from checkpoint containers saves 2 minutes on fast local disks and Amazon EBS disks. The savings is almost 20 minutes when using Azure network storage where the disk caching scheme appears to be much less favorable to STAR's indexing process.

Here, we have presented a novel idea for optimizing cloud deployments using checkpointing to save containers where the applications are already started. Using CRIU for Docker, we can save the container with a preloaded genome for STAR alignment and restore the container from these checkpoint files to any host. We have achieved successful migration of checkpointed containers to different virtual machine instances running on the Amazon and Azure cloud platforms while realizing up to a 3.57x speedup using our approach, saving up to 20 minutes for a single STAR alignment workflow on Azure with network disks. For STAR alignment, it is possible to use a checkpointed container to align multiple sequences at once by retaining the genomic indices in memory. Our approach yields a significant benefit with hot-starting when as few as one or two files are aligned. Additionally, multiple STAR alignment tasks can be computed in parallel using the same genome indices hosted by different processes. For automated schedulers such as Docker Compose [17], “hot-starting” reduces execution time every single time the STAR container is launched. While it is possible to design a workflow to perform all the alignments in a single container first, load-balancing would be made easier by allowing the scheduler to distribute the computation over the cluster as shorter jobs.

There are a few caveats to the hot-start strategy. First, the CRIU tool produces checkpoint files that are Linux kernel version dependent [18]. Restoring a checkpoint on a Docker host in a local cluster or an instance in the cloud backed by a different kernel version would require a kernel-specific checkpoint file that can be created by running the CRIU tool on the node or instance. Second is the requirement for a convenient place in the workflow to insert a pause, checkpoint, and re-start. In the case of STAR, this is provided by a flag that allows the container to keep genomic indices in shell memory between invocations of STAR. For other workflows, one could add a flag to pause the computation where the checkpoint is to be created and a flag to resume the computation afterward. With these straightforward modifications, any workflow could take advantage of checkpointing to avoid repetitive initialization steps. A major advantage of hot-starting is that it does not require extensive knowledge of the underlying code to optimize performance. While it may be more efficient to simply rewrite the code to eliminate repetitive steps, this is not always feasible, especially for academic or poorly documented legacy software. Hot-starting from pre-initialized containers represents a novel and unexplored approach to speeding up bioinformatics workflows deployed on the cloud or local servers.

## Methods

CRIU is a Linux software tool that freezes a running application and saves it as a collection of files to disk [12]. The application can later be restored on the same host or on a different host. Docker currently integrates CRIU as an experimental checkpoint subcommand that saves the state of processes to a collection of files on disk. The checkpointing command has been used to migrate containers from the source host to a target host when the resources of the source are limited [19], for fault tolerance purposes [20], and to provide highly available and scalable microservices [21].

### Cloud configurations tested

In our experiments, we deployed our containers on instances from two cloud platforms: Microsoft Azure and AWS. Ubuntu 16.04 was the host operating system in all of our tests. Specifically, we used Ubuntu server 16.04 LTS with Ubuntu Kernel version 4.4.0–28-generic and CRIU version 3.1 “Graphene Swift” in our empirical studies on Microsoft Azure. We used Ubuntu 16.04.03 LTS with Kernel version 4.4.0–1022-aws and CRIU version 3.1 “Graphene Swift” in our empirical studies on AWS. Testing was conducted using a standard DS13 v2 instance with eight virtual central processing units (CPUs) and 56 Gb memory on Azure and a m4.4xlarge instance with 16 virtual CPUs and 64 Gb memory on AWS. As disk input/output (I/O) is an important factor in the efficiency of CRIU restoration and the generation of indices without CRIU, instances were tested using both network-based disks (EBS for AWS and Microsoft Azure File Storage for Azure) and locally attached disks.

### Creating hot-start containers

We installed CRIU on the host Ubuntu system. Docker Community Edition, which includes the experimental checkpointing tool, was then installed. The STAR binary was compiled from source [22] using Ubuntu 16.04 and g++ and then copied into a clean Ubuntu 16.04 container with no intermediate build files. The build code and Docker files are available from [23]. To create the checkpoint, STAR was launched with the *genomeLoad* flag set to *LoadAndKeep*. This keeps the indices in shared memory after STAR exits. To trap the container in this state, we launched STAR using a parent shell script that did not exit and checkpointed the container after STAR exited. This results in the generation of checkpoint files that store the state of the hot-start container. Because different Linux kernel versions are used on AWS and Azure, we created separate hot-start containers for each cloud.

### Comparing hot-start containers and standard cold-start containers

The paired-end fastq files were 9 Gb in size comprising 22,935,521 reads. Times were recorded for the generation of bam files (binary version of sequence alignment data) generated by STAR in the standard container and using STAR with the hot-start container. Times include the time required to restore the hot-start container from the checkpointed files.

## Supplementary Material

GIGA-D-17-00326_Original_Submission.pdf

GIGA-D-17-00326_Revision_1.pdf

GIGA-D-17-00326_Revision_2.pdf

Response_to_Reviewer_Comments_Original_Submission.pdf

Response_to_Reviewer_Comments_Revision1.pdf

Reviewer_1_Report_(Original_Submission) -- Mark Fernandes 1/15/2018 Reviewed

Reviewer_2_Report_(Original_Submission) -- Francois Moreews 1/23/2018 Reviewed

Reviewer_3_Report_(Original_Submission) -- Björn Grüning 2/12/2018 Reviewed

Reviewer_3_Report_(Revision_1) -- Björn Grüning 5/13/2018 Reviewed

Additional Files

## Availability and requirements

**Project name:** Hot-starting software container for STAR Alignment

**Project homepage:** https://github.com/biodepot/Hotstarting-For-STAR-Alignment

**DockerHub URL:** https://hub.docker.com/r/biodepot/star-for-criu/

**Operating system:** Ubuntu 16.04

**Programming language:** Shell

**Other requirements:** Docker API version 1.25 or higher, CRIU 2.0 or later, Linux kernel v3.11 or higher are required.

**License:** MIT License.

**RRID on SciCrunch.org**: RRID:SCR_016294

## Availability of supporting data

The fastq files used in our tests were generated by Himes et al. and are publicly available from GEO with accession number GSE52778 (https://www.ncbi.nlm.nih.gov/geo/query/acc.cgi?acc=GSE52778). Snapshots of the code and base containers are hosted in the *GigaScience* GigaDB repository [24].

## Additional files

**Additional File 1:** User manual for “Hot-starting software container for STAR Alignment”

**Additional File 2:** Raw data, average running time, standard error and standard deviation across five runs of STAR alignment with checkpoint and without checkpoint. Our empirical experiments were performed on local and network disks using Amazon Web Services (AWS) and Microsoft Azure.

## Abbreviations

AWS: Amazon Web Services; CPU: central processing unit; CRIU: Checkpoint/Restore In Userspace; EBS: Elastic Block Store; RNA-seq: RNA sequencing; STAR: spliced transcripts alignment to a reference.
