# Supplementary material for: Hot-starting software containers for STAR aligner
Source: Gigascience. 2018 Jul 31;7(8):giy092. doi: 10.1093/gigascience/giy092 (PMC6131214; doi:10.1093/gigascience/giy092)
Supplement: GIGA-D-17-00326_Revision_1.pdf [file giy092_giga-d-17-00326_revision_1.pdf]

GigaScience
Hot-starting software containers for STAR aligner
--Manuscript Draft--

**Manuscript Number:**	GIGA-D-17-00326R1
**Full Title:**	Hot-starting software containers for STAR aligner
**Article Type:**	Technical Note
**Funding Information:**	National Institutes of Health (U54HL127624) — Not applicable<br>AMEDD Advanced Medical Technology Initiative — Not applicable<br>National Institutes of Health (R01GM126019) — Dr. Ka Yee Yeung

Abstract:

Background: Using software containers has become standard practice to reproducibly deploy and execute biomedical workflows on the cloud. However, some applications which contain time-consuming initialization steps will produce unnecessary costs for repeated executions.

Findings: We demonstrate that hot-starting, from containers that have been frozen after the application has already begun execution, can speed up bioinformatics workflows by avoiding repetitive initialization steps. We use an open source tool called Checkpoint and Restore in Userspace (CRIU) to save the state of the containers as a collection of checkpoint files on disk after it has read in the indices. The resulting checkpoint files are migrated to the host and CRIU is used to regenerate the containers in that ready-to-run hot-start state. As a proof-of-concept example, we create a hot-start container for the STAR aligner and deploy this container to align RNA sequencing data. We compare the performance of the alignment step with and without checkpoints on cloud platforms using local and network disks.

Conclusions: We demonstrate that hot-starting Docker containers from snapshots taken after repetitive initialization steps are completed, significantly speeds up the execution of the STAR aligner on all experimental platforms, including Amazon Web Services (AWS), Microsoft Azure and local virtual machines. Our method can be potentially employed in other bioinformatics applications in which a checkpoint can be inserted after a repetitive initialization phase.

**Corresponding Author:**	Ka Yee Yeung, Ph.D.<br>University of Washington Tacoma<br>UNITED STATES
**Corresponding Author Secondary Information:**	
**Corresponding Author's Institution:**	University of Washington Tacoma
**Corresponding Author's Secondary Institution:**	
**First Author:**	Pai Zhang
**First Author Secondary Information:**	
**Order of Authors:**	Pai Zhang<br>Ling-Hong Hung, Ph.D.<br>Wes Lloyd, Ph.D.<br>Ka Yee Yeung, Ph.D.
**Order of Authors Secondary Information:**	

**Response to Reviewers:**	Title: Hot-starting software containers for STAR aligner<br>Manuscript ID: GIGA-D-17-00326

Powered by Editorial Manager® and ProduXion Manager® from Aries Systems Corporation

Note that we changed the title of the manuscript in response to Reviewer 3.

RESPONSE TO REVIEWERS
The authors greatly appreciate the critique from all three reviewers. We addressed all the suggestions in the reports. The specific responses are shown below, with our responses in italic Times New Roman.

Reviewer #1:
This paper introduces the concept that Cloud computing and Containers offer new routes to pipeline optimisation. I like the use of multiple Cloud providers which highlights issues with optimisation for different network filesystems.
It would have been interesting to have a comparison with a typical HPC approach e.g. saving the pre-computed indices to a parallel file system such as Lustre to highlight the optimisation wrt retaining indices in memory.
With respect to HPC CRIU may also have some utility there e.g. with Singularity containers which are often accepted by Cluster admins compared to Docker.

Our response:
While our manuscript focuses on Docker containers, we appreciate the feedback to discuss other container technology. In response to your comments, we added a sentence and two references on Singularity containers in the manuscript in the first paragraph under “Background”. Specifically, the following references are added:
5. Kurtzer GM, Sochat V and Bauer MW. Singularity: Scientific containers for mobility of compute. PLoS One. 2017;12 5:e0177459. doi:10.1371/journal.pone.0177459.
6. Sochat VV, Prybol CJ and Kurtzer GM. Enhancing reproducibility in scientific computing: Metrics and registry for Singularity containers. PLoS One. 2017;12 11:e0188511. doi:10.1371/journal.pone.0188511.

I visited the web resources for the software and found them fairly comprehensive and active.

Our response:
Thank you for checking out our web resources and for your feedback.

Reviewer #2:
The authors propose a new technique to optimise the cost and execution time of data analysis using containers in batch processing mode. This technique relies on an existing tool named CRIU (Checkpoint and Restore in Userspace) that allows to "freeze" the state of a linux process and acts as a "snapshot" including RAM state. CRIU persists a state of a running application to a hard drive as a collection of files.

The innovation described in the paper consists in the application of this checkpoint technique to data processing tools executed in Docker containers. The detailed benchmark is based on the STAR aligner, a sequence alignment tool used for high-throughput RNA-seq data.
In the use case described in the paper, the container executing the software is frozen after the reference index creation, a costly initial step. Then, the "snapshot" container can be reused in a loop, iterating the whole data collection, but without the cost of the index creation. Then , the benchmark shows that the method reduces the aligner execution time.
Even if CRIU is already available as an experimental feature in Docker, Openvz and LXC, the described work brings something new. Based on our current knowledge and referenced publications, the idea of using "frozen containers" to reduce the time and cost of execution does not seem to have ever been published before.

The proposition of using these "Hot-starting software containers" to improve the performance of bioinformatics data analysis looks promising especially combined with data parallelisation.
It can have a strong impact on cost and execution time of high throughput data analysis using containers like Docker or LXC, especially on a commercial cloud.
We suggest to precise that the described method is especially interesting in the context of "heterogeneous" and "legacy" software integration which is not covered in the

Powered by Editorial Manager® and ProduXion Manager® from Aries Systems Corporation

paper.
Indeed, a classical approach to optimise STAR is its direct code modification. A coded new feature that allows to reuse and existing persisted index can produce a similar optimisation.
The authors may explain that this naive solution is not always possible because bioinformaticians are reusing a lot of tools considered as "black boxes" and sometimes the tools are simply not maintained any more. A bioinformatics workflow generally embeds external legacy software building blocks developed by multiple authors and the workflow developer is often not the author of the building blocks.
In these cases, common in bioinformatics, the "Hot-starting software containers" optimisation technique can be very useful.
I suggest also to put emphasis on the fact that the technique is as well important for speed-up workflows, not only for cost. Moreover, containers can be used on local PC and not only on clouds.

Our response:
We greatly appreciate the suggested elaborations.

We made the following revisions in the manuscript to focus on speed-up instead of costs:
- Abstract (page 2). “We demonstrate that hot-starting, from containers that have been frozen after the application has already begun execution, can speed up bioinformatics workflows by avoiding repetitive initialization steps.”
- Abstract (page 2). “We demonstrate that hot-starting Docker containers from snapshots taken after repetitive initialization steps are completed, significantly speeds up the execution of the STAR aligner on all experimental platforms …”
- Before “Methods” on page 6. “Hot-starting from pre-initialized containers represents a novel and unexplored approach to speeding up bioinformatics workflows deployed on the cloud or local servers. ”

We also added a few sentences in the paragraph before “Methods” on page 6 to discuss the merits of our hot-starting container approach in contrast to code optimization as suggested by the reviewer. Specifically,
“A major advantage of hot-starting is that it does not require extensive knowledge of the underlying code to optimize performance. While it may be more efficient to simply re-write the code to eliminate repetitive steps – this is not always feasible especially for academic or poorly documented legacy software. ”

Reviewer #3:
In there manuscript called “Hot-starting software containers for bioinformatics analyses" Pai Zhang and colleagues describing an idea about hot-starting containers and providing some benchmarks with the claim that this could speed up calculations, potentially many, and improving overall performance. This was demonstrated with the well known STAR software for mapping reads.

While the idea on a first glance looks super cool and the graphs promising some major performance difference I have some major concerns and questions.

The authors compare a system where a container is generating the index on the fly, with a system that has a pre-build index in memory. But in reality people do not generate the index on the fly but using pre-build indices that are mounted from external source into the container. It would be interesting to see how much faster this approach is, if the index does not need to be generated but can be mounted as is into the container. Please elaborate on the performance difference between "STAR reads the index into memory" and "mmap reads the memory-container dump".
Pre-generated indices are provided by a lot of different community projects and can be even mounted into containers. Please discuss if the performance gain of your approach is worth the extra steps and the loss in reproducibility and usability.

Our response:
We would like to clarify that we did not generate indices on the fly, and the testing was done with pre-generated indices. We understand and apologize for the loose wording in the original manuscript where we refer to generation of indices. All the experiments were done with pre-generated indices. The initialization step that we are referring to,

Powered by Editorial Manager® and ProduXion Manager® from Aries Systems Corporation

that we avoid, is the reading of the indices into memory. We have made extensive corrections throughout the abstract, manuscript and figure legends to make this clear.

Specifically, we clarified this point in the following places in the manuscript:
- Abstract (page 2) Findings: “We use an open source tool called Checkpoint and Restore in Userspace (CRIU) to save the state of the containers as a collection of checkpoint files on disk after it has read in the indices.”
- “Our Approach” on page 4: “For STAR, the process of reading in the indices is a slow process and STAR has an option of keeping the indices in memory after they have been generated so that subsequent sequence alignments do not have to repeat the step of reading the indices. We used the CRIU tool to create checkpoints after the indices have been read. “

Assuming hot-starting a container in comparison to using a pre-build index is still faster I would like to see a small discussion about how this compares in price and efficiency. Because with this approach a researcher still needs to transfer and store the memory dump in the cloud - and storage in the cloud is not cheap.

Our response:
The reviewer is absolutely correct. We have not taken into account the charges for long-term storage onto the cloud for different storage types. We have removed the language referring to cost savings and focus on speed-up which we do directly benchmark.
- Abstract (page 2). “We demonstrate that hot-starting, from containers that have been frozen after the application has already begun execution, can speed up bioinformatics workflows by avoiding repetitive initialization steps.”
- Abstract (page 2). “We demonstrate that hot-starting Docker containers from snapshots taken after repetitive initialization steps are completed, significantly speeds up the execution of the STAR aligner on all experimental platforms …”
- Before “Methods” on page 6. “Hot-starting from pre-initialized containers represents a novel and unexplored approach to speeding up bioinformatics workflows deployed on the cloud or local servers. ”

In the last sentence it was mentioned that these snapshots are more or less not transferable to arbitrary hosts because of the different kernel versions. This is a major drawback of this approach and should be more prominently discussed. How stable is the interface between kernel versions or operating systems? How is reproducibility guaranteed? What can happen if I choose the wrong memory dump?

Our response:
This is true of any containerized application. We rely on Docker to handle the low-level interactions with the kernel and managing reproducibility. We have added more text to the Discussion section to elaborate on this point. Specifically, we added the following sentences on page 6:
“There are several caveats to the hot-start strategy. One is that the CRIU tool is Linux kernel version dependent [18]. Checkpoint files are not portable among hosts where different versions of the Linux kernel are used. However, this is an implicit feature for any container workflow: the reproducibility of components outside the container depend upon a platform-specific implementation of the containerization software. ”

To address your point about how reproducibility, we removed the word “guarantee” in the manuscript. Specifically, on page 3
“Other container technologies such as Singularity containers have also been proposed to enhance mobility and reproducibility of computational science [5, 6]. Thus, containerization enhances the reproducibility of bioinformatics workflows [7-9]. ”

How many times was the experiment repeated to produce Figure 2? A standard deviation is missing in this figure.

Our response:
The experiment results shown in Figure 2 are repeated 5 times. We clarified this point in both the main text and caption for Figure 2.

Powered by Editorial Manager® and ProduXion Manager® from Aries Systems Corporation

	In addition, we added Additional File 2 that shows the raw results, average, standard deviation and standard errors of alignment time (seconds) for each of the 4 settings (AWS with local disk, AWS EBS, Azure local host, Azure File Storage). We did not add the error bars to Figure 2 because the standard deviations and standard errors for the experiments across the 5 runs are very small compared to the total alignment time, and hence, cannot be shown clearly in the figure.<br><br>The authors claim that this approach is usable by other bioinformatics software. I would like to see at least 2-3 other examples where this speedup is archived. If not please adopt the title and don't make generalize claims.<br><br>Our response:<br>The reviewer's point is well-taken. We have modified the title of the manuscript to be "Hot-starting software containers for STAR aligner" so as to be more specific.<br><br>The authors using the term containers, but only mentioned Docker in the manuscript. Is the same technique possible using other container technologies? Singularity or rkt for example?<br><br>Our response:<br>Our manuscript focuses on Docker containers. We added a sentence and two references on Singularity containers in the manuscript in the first paragraph under "Background" to acknowledge that there are other container technologies. Specifically, the following references are added:<br>5. Kurtzer GM, Sochat V and Bauer MW. Singularity: Scientific containers for mobility of compute. PLoS One. 2017;12 5:e0177459. doi:10.1371/journal.pone.0177459.<br>6. Sochat VV, Prybol CJ and Kurtzer GM. Enhancing reproducibility in scientific computing: Metrics and registry for Singularity containers. PLoS One. 2017;12 11:e0188511. doi:10.1371/journal.pone.0188511.<br><br>Figure 2 has a bad quality this should be improved.<br><br>Our response:<br>Thank you for the suggestion. We have submitted higher resolution figures for both Figure 1 and Figure 2 in this revision.<br><br>A citation for the paragraph "Thus, containerization enhances ..." would be nice. There is a lot of literature and big communities in bioinformatics that have studied this topic already.<br><br>Our response:<br>In response to this comment, three references are added to support this statement in the first paragraph under "Background" on page 1. Specifically, the following references are added:<br><br>7. Schulz WL, Durant TJ, Siddon AJ and Torres R. Use of application containers and workflows for genomic data analysis. Journal of pathology informatics. 2016;7:53. doi:10.4103/2153-3539.197197.<br>8. Silver A. Software simplified: Containerization technology takes the hassle out of setting up software and can boost the reproducibility of data-driven research. Nature. 2017;546:173-4.<br>9. Piccolo SR and Frampton MB. Tools and techniques for computational reproducibility. GigaScience. 2016;5 1:30. doi:10.1186/s13742-016-0135-4.
**Additional Information:**	
**Question**	**Response**
Are you submitting this manuscript to a special series or article collection?	No
**Experimental design and statistics**<br><br><br>Full details of the experimental design and	Yes

statistical methods used should be given in the Methods section, as detailed in our Minimum Standards Reporting Checklist. Information essential to interpreting the data presented should be made available in the figure legends.<br><br>Have you included all the information requested in your manuscript?	
**Resources**<br><br>A description of all resources used, including antibodies, cell lines, animals and software tools, with enough information to allow them to be uniquely identified, should be included in the Methods section. Authors are strongly encouraged to cite Research Resource Identifiers (RRIDs) for antibodies, model organisms and tools, where possible.<br><br>Have you included the information requested as detailed in our Minimum Standards Reporting Checklist?	Yes
**Availability of data and materials**<br><br>All datasets and code on which the conclusions of the paper rely must be either included in your submission or deposited in publicly available repositories (where available and ethically appropriate), referencing such data using a unique identifier in the references and in the "Availability of Data and Materials" section of your manuscript.<br><br>Have you have met the above requirement as detailed in our Minimum Standards Reporting Checklist?	Yes

Manuscript

Click here to download Manuscript CRIU docker For GIgaScience-noFig-submit.pdf

Hot-starting software containers for STAR aligner

Pai Zhang
Institute of Technology, University of Washington, Tacoma, Washington 98402, USA.
Email: paizhang@uw.edu

Ling-Hong Hung
Institute of Technology, University of Washington, Tacoma, Washington 98402, USA.
Email: lhhung@uw.edu

Wes Lloyd
Institute of Technology, University of Washington, Tacoma, Washington 98402, USA.
Email: wlloyd@uw.edu

Ka Yee Yeung
Institute of Technology, University of Washington, Tacoma, Washington 98402, USA.
Email: kayee@uw.edu

Correspondence should be addressed to K.Y.Y. (kayee@uw.edu)

ABSTRACT

Background: Using software containers has become standard practice to reproducibly deploy and execute biomedical workflows on the cloud. However, some applications which contain time-consuming initialization steps will produce unnecessary costs for repeated executions.

Findings: We demonstrate that hot-starting, from containers that have been frozen after the application has already begun execution, can speed up bioinformatics workflows by avoiding repetitive initialization steps. We use an open source tool called Checkpoint and Restore in Userspace (CRIU) to save the state of the containers as a collection of checkpoint files on disk after it has read in the indices. The resulting checkpoint files are migrated to the host and CRIU is used to regenerate the containers in that ready-to-run hot-start state. As a proof-of-concept example, we create a hot-start container for the STAR aligner and deploy this container to align RNA sequencing data. We compare the performance of the alignment step with and without checkpoints on cloud platforms using local and network disks.

Conclusions: We demonstrate that hot-starting Docker containers from snapshots taken after repetitive initialization steps are completed, significantly speeds up the execution of the STAR aligner on all experimental platforms, including Amazon Web Services (AWS), Microsoft Azure and local virtual machines. Our method can be potentially employed in other bioinformatics applications in which a checkpoint can be inserted after a repetitive initialization phase.

Keywords: software container, reproducibility of research, cloud computing

Findings

Background

With the availability of high-throughput next generation sequencing technologies and the subsequent explosion of big biomedical data, the processing of biomedical big data has become a major challenge. Cloud computing plays an important role in addressing this challenge by offering massive scalable computing and storage, data sharing and on-demand access to resources and applications [1, 2]. The National Institutes of Health is launching a Data Commons Pilot Phase to provide access and storage of biomedical data and bioinformatics tools on the cloud [3]. Additionally, software containers have become increasingly popular for deploying bioinformatics workflows on the cloud. Docker [4], an open source project, has become the *de facto* standard for container software. Docker packages executables with all the necessary software dependencies ensuring that the same software environment is replicated regardless of the host hardware and operating system. Other container technologies such as Singularity containers have also been proposed to enhance mobility and reproducibility of computational science [5, 6]. Thus, containerization enhances the reproducibility of bioinformatics workflows [7-9]. In the context of cloud computing, the utility of containers comes from the ease in which a virtual cloud cluster can be rapidly provisioned with all of the necessary dependencies for a complicated workflow by simply downloading a set of containers, each of which take a few seconds to spin up. Recently, Vivian *et al*. processed over 20,000 RNA sequencing (RNA-seq) samples from the Cancer Genome Atlas (TCGA) using Docker containers on the cloud [10]. Tatlow *et al.* used software containers to study the performance and cost profiles of different cloud-based configurations in processing RNA-seq data from public cancer compendia [11].

When containers are deployed, applications are launched *de novo* each time the container is spun up. This means that any initial preparatory steps are repeated each time the container is used. For tasks such as the alignment of reads, these initial steps can be quite substantive as large sets of indices need to be read before alignments can begin. In an automated large-scale deployment, these steps are replicated many times. It would be far more efficient if one could "*checkpoint*" and save containers in states where the application has already completed the initialization steps so as to avoid unnecessary repetitions. One could then "*hot-start*" workflows from these checkpoints. This is analogous to hot-start PCR where all the necessary reagents are pre-mixed awaiting only the addition of the template.

Our approach

Our key idea is to save and restore memory states in software containers using the Checkpoint Restore in Userspace (CRIU) tool. CRIU freezes a running container and saves the checkpoint as a collection of files on disk [12]. These files can subsequently be used to restore and resume the application from that checkpoint. CRIU was originally developed for Linux, but has recently become available for Docker [13]. While it is possible to stop Docker containers with native Docker commands, this process does not preserve the memory state. Although re-starting from a ready-to-go state is an intuitive application of checkpointing, we have been unable to find any previous description of using checkpointing as a general method for improving the efficiency of container deployments.

We demonstrate that hot-starting from a saved container checkpoint can significantly reduce the execution time using the STAR aligner [14, 15] for RNA-seq data analyses. We choose STAR as a proof-of-concept example because it has such a slow initialization step that it includes an option to retain indices in memory for use when aligning many different files. However, our idea of using checkpoints has broad applications in optimizing performance using software containers on the cloud *when performing any bioinformatics task where a pause could be inserted to capture a re-usable state*.

The STAR aligner consists of several steps. Indices are generated from the reference genome. This is typically done just once using the latest version of the reference. The indices are read in and then read sequences from a specific experiment sample are mapped to the reference genome. For STAR, the process of reading in the indices is a slow process and STAR has an option of keeping the indices in memory after they have been generated so that subsequent sequence alignments do not have to repeat the step of reading the indices. We used the CRIU tool to create checkpoints after the indices have been read. Instead of launching a new container and starting STAR from scratch, we restore the container state using CRIU and resume running STAR after it has loaded the indices. **Figure 1** shows an overview of our approach with and without using checkpoints.

Testing

To test the checkpointing methodology, we used RNA-seq data generated by Himes *et al*. which measure the gene expression changes in human airway smooth muscle cells in response to asthma medications [16]. We compared the time required to align the sequences with a normal

container where STAR starts from scratch, and the time required when hot-starting from a container checkpoint where STAR has already generated indices. We performed empirical studies on multiple cloud platforms including Amazon Web Services (AWS) and Microsoft Azure, using both local and networked disks. On AWS, we compared performance with data stored on the local host versus Amazon Elastic Block Store (EBS). On Microsoft Azure, we compared the performance with data stored on the local host versus Azure File Storage. Please refer to the Online Methods for details of our experimental setup. Our empirical results are shown in **Figure 2**.

Figure 2 shows that the STAR aligner with checkpointing reduces the execution time compared to STAR without checkpointing. The average running time over five separate runs are shown. The raw data, average running time and standard deviation across the five runs are available as Additional File 2. On AWS, we observed a 1.89x speedup with data stored on the local disk and 1.42x speedup with data on a network disk (Amazon EBS). On Microsoft Azure, we achieved a 1.34x speedup with data stored on the local disk, and 3.57x speedup with data on Azure File Storage. With respect to execution time, we show that hot-starting from checkpoint containers save 2 minutes on fast local disks and Amazon EBS disks. The saving is almost 20 minutes when using Azure network storage where the disk caching scheme appears to be much less favorable to STAR's indexing process.

In this article, we have presented a novel idea for optimizing cloud deployments using checkpointing to save containers where the applications are already started. Using CRIU for Docker, we can save the container with a preloaded genome for STAR alignment and restore the container from these checkpoint files to any host. We have achieved successful migration of checkpointed containers to different virtual machine instances running on the Amazon and Azure cloud platforms while realizing up to a 3.57x speedup using our approach saving up to 20 minutes for a single STAR alignment workflow on Azure with network disks. For STAR alignment, it is possible to use a checkpointed container to align multiple sequences at once by retaining the genomic indices in memory. Our approach yields a significant benefit with hot-starting when as few as one or two files are aligned. Additionally, multiple STAR alignment tasks can be computed in parallel using the same genome indices hosted by different processes. For automated schedulers such as Docker Compose [17], "hot-starting" reduces execution time every single time the STAR container is launched. While it is possible to design a workflow to perform all the alignments in a single container first, load-balancing would be

made easier by allowing the scheduler to distribute the computation over the cluster as shorter jobs.

There are several caveats to the hot-start strategy. One is that the CRIU tool is Linux kernel version dependent [18]. Checkpoint files are not portable among hosts where different versions of the Linux kernel are used. However, this is an implicit feature for any container workflow: the reproducibility of components outside the container depend upon a platform-specific implementation of the containerization software. Another requirement for our hot-start strategy is that there is a convenient place in the workflow to insert a pause, checkpoint and re-start. In the case of STAR, this is provided by a flag that allows the container to keep genomic indices in shell memory between invocations of STAR. For other workflows, one could add a flag to pause the computation where the checkpoint is to be created, and a flag to resume the computation afterwards. With these straightforward modifications, any workflow could take advantage of checkpointing to avoid repetitive initialization steps. A major advantage of hot-starting is that it does not require extensive knowledge of the underlying code to optimize performance. While it may be more efficient to simply re-write the code to eliminate repetitive steps – this is not always feasible especially for academic or poorly documented legacy software. Hot-starting from pre-initialized containers represents a novel and unexplored approach to speeding up bioinformatics workflows deployed on the cloud or local servers.

METHODS

CRIU. CRIU (Checkpoint/Restore In Userspace) is a Linux software tool that freezes a running application and saves it as a collection of files to disk [12]. The application can later be restored on the same or on a different host. Docker currently integrates CRIU as an experimental checkpoint sub-command that saves the state of processes to a collection of files on disk. The checkpointing command has been used to migrate containers from the source host to a target host when the resources of the source are limited [19], for fault tolerance purposes [20], and to provide highly available and scalable micro-services [21].

Cloud configurations tested

In our experiments, we deployed our containers on instances from two cloud platforms: Microsoft Azure and Amazon Web Services (AWS). Ubuntu 16.04 was the host operating system in all our tests. Specifically, we used Ubuntu server 16.04 LTS with Ubuntu Kernel version 4.4.0-28-generic and CRIU version 3.1 "Graphene Swift" in our empirical studies on

Microsoft Azure. We used Ubuntu 16.04.03 LTS with Kernel version 4.4.0-1022-aws and CRIU version 3.1 "Graphene Swift" in our empirical studies on AWS. Testing was conducted using a standard DS13 v2 instance with 8 virtual CPUs and 56 Gb memory on Azure and a m4.4xlarge instance with 16 virtual CPUs and 64 GB memory on AWS. As disk I/O is an important factor in the efficiency of CRIU restoration and the generation of indices without CRIU, instances were tested using both network based disks (EBS for AWS and Microsoft Azure File Storage for Azure) and locally attached disks.

Creating hot-start containers

We installed CRIU on the host Ubuntu system. Docker Community Edition (Docker CE), which includes the experimental checkpointing tool, was then installed. The STAR binary was compiled from source (https://github.com/alexdobin/STAR) using Ubuntu 16.04 and g++ and then copied into a clean Ubuntu 16.04 container with no intermediate build files. The build code and Dockerfiles are available from https://github.com/BioDepot/ubuntu-star. To create the checkpoint, STAR was launched with the *genomeLoad* flag set to *LoadAndKeep*. This keeps the indices in shared memory after STAR exits. To trap the container in this state, we launched STAR using a parent shell script that did not exit, and checkpointed the container after STAR exited. This results in the generation of checkpoint files that store the state of the hot-start container. Due to different Linux kernel versions being used on AWS and Azure, we created separate hot-start containers for each cloud.

Comparing hot-start containers and standard cold-start containers

The paired-end fastq files were 9 Gb in size comprising 22,935,521 reads. Times were recorded for the generation of aligned BAM files using STAR in the standard container and using STAR with the hot-start container. Times include the time required to restore the hot-start container from the checkpointed files.

AVAILABILITY AND REQUIREMENTS

Project name: Hot-starting software container for STAR Alignment

Project homepage: https://github.com/paizhang/Hotstarting-For-STAR-Alignment

DockerHub URL: https://hub.docker.com/r/biodepot/star-for-criu/

Operating system: Ubuntu 16.04

Programming language: Shell

Other requirements: Docker API version 1.25 or higher, CRIU 2.0 or later, Linux kernel v3.11 or higher are required.

License: MIT License.

AVAILABILITY OF SUPPORTING DATA

The fastq files used in our tests were generated by Himes et al. and are publicly available from GEO with accession number GSE52778 (https://www.ncbi.nlm.nih.gov/geo/query/acc.cgi?acc=GSE52778).

ADDITIONAL FILES

Additional File 1: User manual for "Hot-starting software container for STAR Alignment"

Additional File 2: Raw data, average running time, standard error and standard deviation across five runs of STAR alignment with checkpoint and without checkpoint. Our empirical experiments were performed on local and network disks using Amazon Web Services (AWS) and Microsoft Azure.

ABBREVIATIONS:

AWS : Amazon Web Services;

CRIU: Checkpoint/Restore In Userspace;

EBS: Elastic Block Store;

NIH: National Institutes of Health;

STAR: Spliced Transcripts Alignment to a Reference;

TCGA : The Cancer Genome Atlas.

Conflict of interests

The authors declare that they have no competing interests.

AUTHOR CONTRIBUTIONS

P.Z. and L.H.H. implemented the Docker containers. P.Z. conducted the empirical experiments. P.Z., L.H.H. and K.Y.Y. drafted the manuscript. K.Y.Y. and L.H.H. designed the case study. W.L. provided cloud computing expertise. K.Y.Y. coordinated the empirical study. All authors edited the manuscript.

ACKNOWLEDGEMENTS

L.H.H., W.L. and K.Y.Y. are supported by NIH grant R01GM126019. L.H.H. and K.Y.Y. are also supported by NIH grant U54HL127624 and the AMEDD Advanced Medical Technology Initiative. We would like to acknowledge support from the AWS Cloud Credits for Research (to Lloyd and Yeung) and the Microsoft Azure for Research programs (to Hung and Lloyd) for providing cloud computing resources. We would like to acknowledge the Student High Performance Computing Club and the eScience Institute at University of Washington for both technical assistance and computing resources to Pai Zhang.

REFERENCES

1. Calabrese B and Cannataro M. Cloud Computing in Bioinformatics: current solutions and challenges. PeerJ Preprints. 2016;4:e2261v1.

2. Shanahan HP, Owen AM and Harrison AP. Bioinformatics on the cloud computing platform Azure. PLoS One. 2014;9 7:e102642. doi:10.1371/journal.pone.0102642.

3. National Institutes of Health (NIH) Data Commons Pilot. https://commonfund.nih.gov/commons. Accessed April 6, 2018.

4. Docker. https://http://www.docker.com/. Accessed April 6, 2018.

5. Kurtzer GM, Sochat V and Bauer MW. Singularity: Scientific containers for mobility of compute. PLoS One. 2017;12 5:e0177459. doi:10.1371/journal.pone.0177459.

6. Sochat VV, Prybol CJ and Kurtzer GM. Enhancing reproducibility in scientific computing: Metrics and registry for Singularity containers. PLoS One. 2017;12 11:e0188511. doi:10.1371/journal.pone.0188511.

7. Schulz WL, Durant TJ, Siddon AJ and Torres R. Use of application containers and workflows for genomic data analysis. Journal of pathology informatics. 2016;7:53. doi:10.4103/2153-3539.197197.

8. Silver A. Software simplified: Containerization technology takes the hassle out of setting up software and can boost the reproducibility of data-driven research. Nature. 2017;546:173-4.

9. Piccolo SR and Frampton MB. Tools and techniques for computational reproducibility. GigaScience. 2016;5 1:30. doi:10.1186/s13742-016-0135-4.

10. Vivian J, Rao A, Nothaft FA, Ketchum C, Armstrong J, Novak A, et al. Rapid and efficient analysis of 20,000 RNA-seq samples with Toil. bioRxiv. 2016.

11. Tatlow PJ and Piccolo SR. A cloud-based workflow to quantify transcript-expression levels in public cancer compendia. Scientific reports. 2016;6:39259. doi:10.1038/srep39259.

12. Checkpoint Restore in Userspace (CRIU). https://criu.org/Main_Page. Accessed April 6, 2018.

13. CRIU Integration with Docker. https://criu.org/Docker. Accessed April 6, 2018.

14. Dobin A, Davis CA, Schlesinger F, Drenkow J, Zaleski C, Jha S, et al. STAR: ultrafast universal RNA-seq aligner. Bioinformatics. 2013;29 1:15-21. doi:10.1093/bioinformatics/bts635.

15. Dobin A and Gingeras TR. Mapping RNA-seq Reads with STAR. Current protocols in bioinformatics. 2015;51:11 4 1-9. doi:10.1002/0471250953.bi1114s51.

16. Himes BE, Jiang X, Wagner P, Hu R, Wang Q, Klanderman B, et al. RNA-Seq transcriptome profiling identifies CRISPLD2 as a glucocorticoid responsive gene that modulates cytokine function in airway smooth muscle cells. PLoS One. 2014;9 6:e99625. doi:10.1371/journal.pone.0099625.

17. Docker Compose. https://http://www.docker.com/products/docker-compose. Accessed April 6, 2018.

18. CRIU: Linux kernel. https://criu.org/Linux_kernel. Accessed April 6, 2018.

19. Al-Dhuraibi Y, Paraiso F, Djarallah N and Merle P. Autonomic Vertical Elasticity of Docker Containers with ELASTICDOCKER. In: *IEEE 10th International Conference on Cloud Computing* Honolulu, CA, USA, 25-30 June 2017 2017, IEEE.

20. Ismail BI, Goortani EM, Karim MBA, Tat WM, Setapa S, Luke JY, et al. Evaluation of Docker as Edge Computing Platform. In: *IEEE Confernece on Open Systems (ICOS)* Bandar Melaka, Malaysia 24-26 Aug. 2015 2015, IEEE.

21. Chen Y. Checkpoint and Restoration of Micro-service in Docker Containers. In: *Third International Conference on Mechatronics and Industrial Informatics* 2015.

FIGURE CAPTION

Figure 1. An overview of our approach with and without checkpoints. The left panel shows the two steps of the STAR aligner [14, 15] after the generation of indices. The right panel shows our approach using the Checkpoint Restore in Userspace (CRIU) tool that freezes a running container and saves the checkpoint as a collection of files on disk after the genome indices are generated using the reference genome. Our "hot-start" containers use these saved files to restore the application and map the reads from the experimental sample data to the reference.

Figure 2. STAR alignment running time comparison with checkpoint and without checkpoint. The running time is averaged over five runs. We performed our empirical experiments on two cloud platforms: Amazon Web Services (AWS) and Microsoft Azure. Both the Azure File Storage and the Amazon Elastic Block Store (EBS) represent network disks. We observe that our "hot-start" containers (orange and grey bars) provide a major reduction in execution time, especially on local disks.

Figure 1

Click here to download Figure Fig1_Experiment design STAR CRIU.pdf

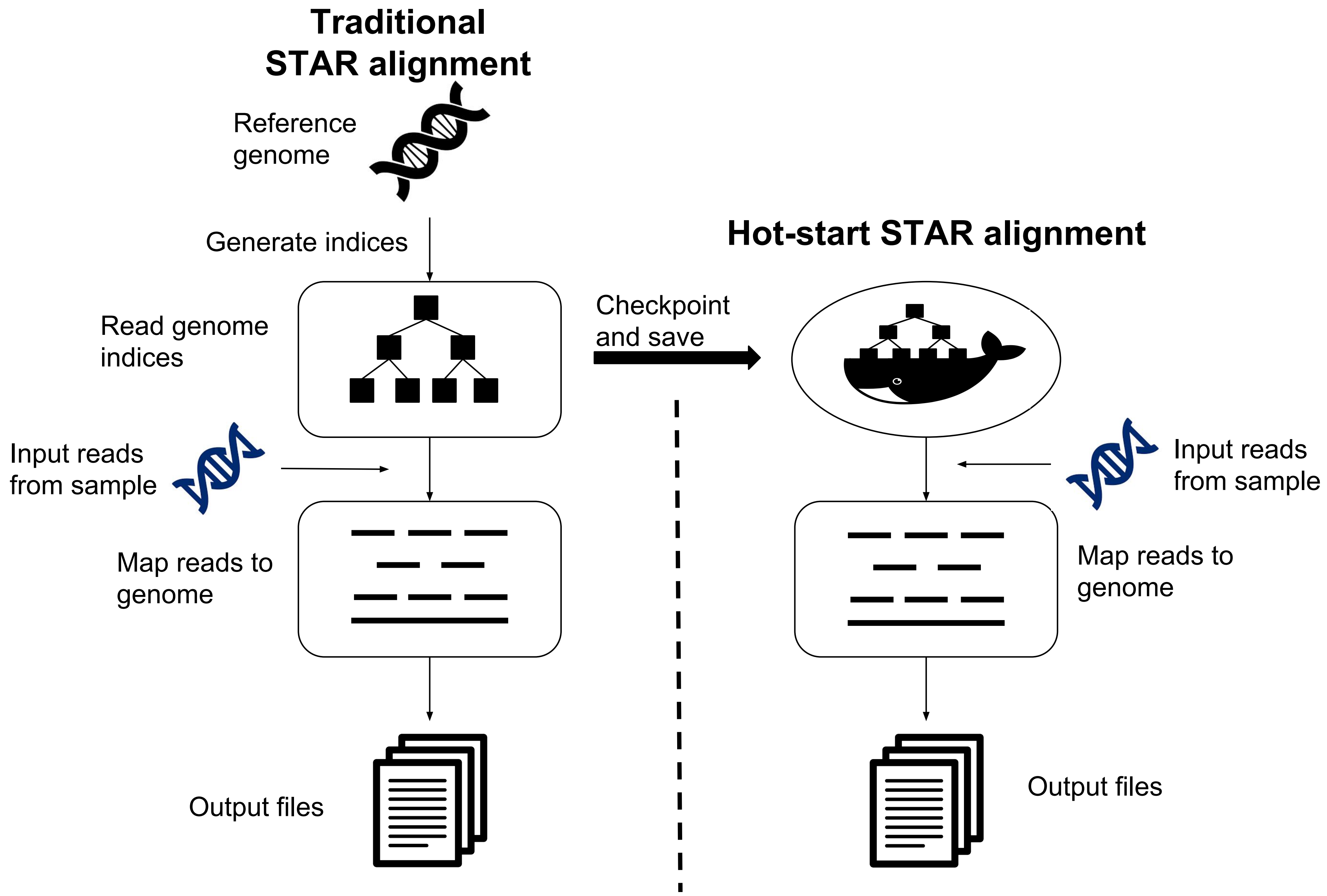

Figure 2 [Click here to download Figure CRIU-figure2.pdf]

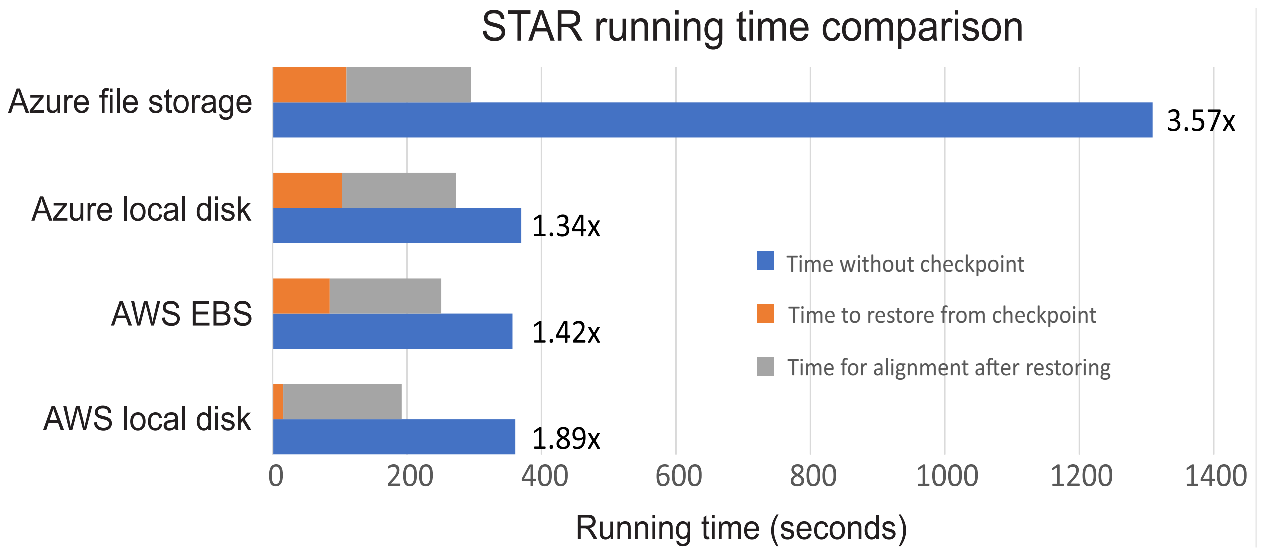

Additional File 1

Click here to access/download
Supplementary Material
User Manual Hot-start Containers New.pdf

Additional File 2

Click here to access/download
Supplementary Material
AdditionalFile2.pdf
