# Supplementary material for: Hot-starting software containers for STAR aligner
Source: Gigascience. 2018 Jul 31;7(8):giy092. doi: 10.1093/gigascience/giy092 (PMC6131214; doi:10.1093/gigascience/giy092)
Supplement: Additional Files [file giy092_supplemental_files.zip › AdditionalFile2.pdf]

Additional File 2: Raw data, average running time, standard error and standard deviation across five runs of STAR alignment with checkpoint and without checkpoint. Our empirical experiments were performed on local and network disks using Amazon Web Services (AWS) and Microsoft Azure.

The color coding in this table are the same as the average results shown in Figure 2.

Running times in seconds

**Azure file storage**	**Run 1**	**Run 2**	**Run 3**	**Run4**	**Run 5**	**Mean**	**Std Dev**	**Std Error**
No checkpoint	1032	1020	1043	1049	1051	1039.0	11.58	5.18
Restore checkpoint	112	109	110	113	110	110.8	1.47	0.66
Alignment	182	190	185	183	186	185.2	2.79	1.25
Hot-start total	294	299	295	296	296	296.0	1.67	0.75

**Azure local disk**	**Run 1**	**Run 2**	**Run 3**	**Run4**	**Run 5**	**Mean**	**Std Dev**	**Std Error**
No checkpoint	365	370	375	371	369	370.0	3.22	1.44
Restore checkpoint	107	102	103	103	105	104.0	1.79	0.80
Alignment	172	169	168	169	173	170.2	1.94	0.87
Hot-start total	279	271	271	272	278	274.2	3.54	1.58

**AWS EBS**	**Run 1**	**Run 2**	**Run 3**	**Run4**	**Run 5**	**Mean**	**Std Dev**	**Std Error**
No checkpoint	395	343	351	345	350	356.8	19.33	8.65
Restore checkpoint	99	90	87	72	77	85.0	9.57	4.28
Alignment	167	167	166	167	164	166.2	1.17	0.52
Hot-start total	266	257	253	239	241	251.2	10.09	4.51

**AWS local disk**	**Run 1**	**Run 2**	**Run 3**	**Run4**	**Run 5**	**Mean**	**Std Dev**	**Std Error**
No checkpoint	359	360	365	359	362	361.0	2.28	1.02
Restore checkpoint	16	16	16	16	17	16.2	0.40	0.18
Alignment	168	170	179	168	193	175.6	9.60	4.30
Hot-start total	184	186	195	184	210	191.8	9.97	4.46
