# Supplementary material for: Hot-starting software containers for STAR aligner
Source: Gigascience. 2018 Jul 31;7(8):giy092. doi: 10.1093/gigascience/giy092 (PMC6131214; doi:10.1093/gigascience/giy092)
Supplement: Additional Files [file giy092_supplemental_files.zip › User Manual Hot-start Containers New.pdf]

Hot-starting Software Container for STAR Alignment

User Manual

Pai Zhang
Institute of Technology, University of Washington, Tacoma, Washington 98402, USA.
Email: paizhang@uw.edu

Ling-Hong Hung
Institute of Technology, University of Washington, Tacoma, Washington 98402, USA.
Email: lhhung@uw.edu

Wes Lloyd
Institute of Technology, University of Washington, Tacoma, Washington 98402, USA.
Email: wlloyd@uw.edu

Ka Yee Yeung
Institute of Technology, University of Washington, Tacoma, Washington 98402, USA.
Email: kayee@uw.edu

This project presents a hot-starting method for STAR Alignment. This approach is to use CRUI (Checkpoint Restore in Userspace) tool to freeze the running container which has the genome indics in the share memory as a collection of files on disk. These checkpoint files are used to restore the hot-start container for STAR mapping step.

Prerequisites

- 64-bit Ubuntu 16.04 Server (Linux kernel v3.11 or newer is required)
- Docker (Docker API version 1.25 or newer required for running CRIU in experimental mode)
- CRIU
- Physical machine or virtual machine with at least 32 GB memery available

Installing

1. Installing Docker on Ubuntu 16.04

   i. First, add the GPG key for the official Docker repository to the system:

      curl -fsSL https://download.docker.com/linux/ubuntu/gpg | sudo apt-key add -

   ii. Add the Docker repository to APT sources:

      sudo add-apt-repository "deb [arch=amd64] https://download.docker.com/linux/ubuntu $(lsb_release -cs) stable"

   iii. update the package database with the Docker packages from the newly added repo:

      sudo apt-get update

   iv. install Docker CE:

      sudo apt-get install -y docker-ce

   v. check whether Docker is running

      sudo systemctl status docker

   If the status of Docker deamon shows "**active (running)**", it means Docker is installed correctly.

2. Install CRIU

   i. Get the CRIU repository from Github:

git clone https://github.com/xemul/criu

ii. Install packages for compiler and C library:

sudo apt-get install build-essential libc6-dev-i386 gcc-multilib

iii. Install packages for protocol buffers: (CRIU use Google Protocol Buffers to read and write image)

sudo apt-get install libprotobuf-dev, libprotobuf-c0-dev, protobuf-c-compiler,protobuf-compiler,python-protobuf,libnet1-dev,pkg-config,libnl-3-dev,python-ipaddr,libbsd,libcap-dev,libaio-dev,python-yaml

iv. Install other packages that are needed for CRIU:

sudo apt-get install --no-install-recommends git build-essential libprotobuf-dev libprotobuf-c0-dev protobuf-c-compiler protobuf-compiler python-protobuf libnl-3-dev libpth-dev pkg-config libcap-dev asciidoc xmlto libnet-dev

v. Install two packages to make install-man work

sudo apt-get install asciidoc xmlto

vi. Building CRIU tool, Enter criu directory and run:

sudo make

vii. Install CRIU, run:

sudo make install

viii. Check whether it works well, it should say "Looks OK" if it is installed correctly.

criu check

ix. Enable docker experimental mode so that we can use **"docker checkpoint"** subcommand:

sudo echo "{\"experimental\": true}" >> /etc/docker/daemon.json

sudo systemctl restart docker

Approach

There are three main steps for this hot-starting methond: generating genome indices, checkpointing container, restoring container and STAR Alignment using restored container.

1. Generating genome indices

   i. Put genome files and RNA-Seq files (FASTQ files) in the same directory, such as: /data.
   ii. Run the docker image ***"biodepot/star-for-criu:latest"*** from DockerHub and execute the run_STAR.sh script inside the container:

      docker run -d --name star -v /data:/data biodepot/star-for-criu:latest /bin/bash /home/run_STAR.sh

   iii. Check the status of STAR alignment:

      docker logs star

   If it says **"Finish loading"**, the genome indices are already in the shared memory.

2. Checkpoint container
   There are two ways to checkpoint the container:

   - Save checkpoint files in default path

     docker checkpoint create star checkpoint-star

   - Save checkpoint files in specific path, such as ***"/home/checkpoint"***

     docker checkpoint create --checkpoint-dir=/home/checkpoint star checkpoint-star

3. Restore container from checkpoint files

   - Restore container from checkpoint files in default path:

     docker start --checkpoint checkpoint-star star-restore

   - Restore container from checkpoint files in specific path:

   i. Create new container but not run it.

      docker create --name star-restore -v /data:/data biodepot/star-for-criu:latest /bin/bash /home/run_STAR.sh

ii. Restore the checkpoint into new container

docker start --checkpoint-dir=/home/checkpoint/CHECKPOINT_ID/checkpoints --checkpoint=checkpoint-star star-restore

CHECKPOINT_ID is the name of the checkpoint folder.

4. STAR Alignment using restored container

   i. Access into the container:

   docker exec -it CONTAINER_ID /bin/bash

   ii. STAR Alignment

   /bin/STAR --runThreadN 8 --genomeDir /data/genome --genomeLoad LoadAndKeep --readFilesIn /data/fastqfiles/SRR1039508_1.fastq /data/fastqfiles/SRR1039508_2.fastq

   We can see the time needed for loading genome in this step is zero because the genome indices is already in the shared memory after we restore the container from checkpoint files.

Deployment and Testing

This method has been tested using a standard DS13 v2 instance with 8 virtual CPUs and 56 Gb memory on Azure and a m4.4xlarge instance with 16 virtual CPUs and 64 Gb memory on AWS. Checkpoint files can be generated on one instance and restored on another instance. The size of generated checkpoint files in this experiment is 27 Gb.

Publication

A pre-print of our manuscript for this project has been submitted to BioRxiv.
The link of the paper:
https://www.biorxiv.org/content/early/2017/10/17/204495

License

This project is licensed under the terms of the MIT license.
