# Supplementary material for: Hot-starting software containers for STAR aligner
Source: Gigascience. 2018 Jul 31;7(8):giy092. doi: 10.1093/gigascience/giy092 (PMC6131214; doi:10.1093/gigascience/giy092)
Supplement: Response_to_Reviewer_Comments_Original_Submission.pdf [file giy092_response_to_reviewer_comments_original_submission.pdf]

Author's Response To Reviewer Comments

Close

Title: Hot-starting software containers for STAR aligner
Manuscript ID: GIGA-D-17-00326

Note that we changed the title of the manuscript in response to Reviewer 3.

RESPONSE TO REVIEWERS
The authors greatly appreciate the critique from all three reviewers. We addressed all the suggestions in the reports. The specific responses are shown below, with our responses in italic Times New Roman.

Reviewer #1:
This paper introduces the concept that Cloud computing and Containers offer new routes to pipeline optimisation. I like the use of multiple Cloud providers which highlights issues with optimisation for different network filesystems.
It would have been interesting to have a comparison with a typical HPC approach e.g. saving the pre-computed indices to a parallel file system such as Lustre to highlight the optimisation wrt retaining indices in memory.
With respect to HPC CRIU may also have some utility there e.g. with Singularity containers which are often accepted by Cluster admins compared to Docker.

Our response:
While our manuscript focuses on Docker containers, we appreciate the feedback to discuss other container technology. In response to your comments, we added a sentence and two references on Singularity containers in the manuscript in the first paragraph under "Background". Specifically, the following references are added:
5. Kurtzer GM, Sochat V and Bauer MW. Singularity: Scientific containers for mobility of compute. PLoS One. 2017;12 5:e0177459. doi:10.1371/journal.pone.0177459.
6. Sochat VV, Prybol CJ and Kurtzer GM. Enhancing reproducibility in scientific computing: Metrics and registry for Singularity containers. PLoS One. 2017;12 11:e0188511. doi:10.1371/journal.pone.0188511.

I visited the web resources for the software and found them fairly comprehensive and active.

Our response:
Thank you for checking out our web resources and for your feedback.

Reviewer #2:
The authors propose a new technique to optimise the cost and execution time of data analysis using containers in batch processing mode. This technique relies on an existing tool named CRIU (Checkpoint and Restore in Userspace) that allows to "freeze" the state of a linux process and acts as a "snapshot" including RAM state. CRIU persists a state of a running application to a hard drive as a collection of files.

The innovation described in the paper consists in the application of this checkpoint

technique to data processing tools executed in Docker containers. The detailed benchmark is based on the STAR aligner, a sequence alignment tool used for high-throughput RNA-seq data.
In the use case described in the paper, the container executing the software is frozen after the reference index creation, a costly initial step. Then, the "snapshot" container can be reused in a loop, iterating the whole data collection, but without the cost of the index creation. Then , the benchmark shows that the method reduces the aligner execution time. Even if CRIU is already available as an experimental feature in Docker, Openvz and LXC, the described work brings something new. Based on our current knowledge and referenced publications, the idea of using "frozen containers" to reduce the time and cost of execution does not seem to have ever been published before.

The proposition of using these "Hot-starting software containers" to improve the performance of bioinformatics data analysis looks promising especially combined with data parallelisation.
It can have a strong impact on cost and execution time of high throughput data analysis using containers like Docker or LXC, especially on a commercial cloud.
We suggest to precise that the described method is especially interesting in the context of "heterogeneous" and "legacy" software integration which is not covered in the paper.
Indeed, a classical approach to optimise STAR is its direct code modification. A coded new feature that allows to reuse and existing persisted index can produce a similar optimisation.
The authors may explain that this naive solution is not always possible because bioinformaticians are reusing a lot of tools considered as "black boxes" and sometimes the tools are simply not maintained any more. A bioinformatics workflow generally embeds external legacy software building blocks developed by multiple authors and the workflow developer is often not the author of the building blocks.
In these cases, common in bioinformatics, the "Hot-starting software containers" optimisation technique can be very useful.
I suggest also to put emphasis on the fact that the technique is as well important for speed-up workflows, not only for cost. Moreover, containers can be used on local PC and not only on clouds.

Our response:
We greatly appreciate the suggested elaborations.

We made the following revisions in the manuscript to focus on speed-up instead of costs:
● Abstract (page 2). “We demonstrate that hot-starting, from containers that have been frozen after the application has already begun execution, can speed up bioinformatics workflows by avoiding repetitive initialization steps.”
● Abstract (page 2). “We demonstrate that hot-starting Docker containers from snapshots taken after repetitive initialization steps are completed, significantly speeds up the execution of the STAR aligner on all experimental platforms …”
● Before “Methods” on page 6. “Hot-starting from pre-initialized containers represents a novel and unexplored approach to speeding up bioinformatics workflows deployed on the cloud or local servers. ”

We also added a few sentences in the paragraph before “Methods” on page 6 to discuss the merits of our hot-starting container approach in contrast to code optimization as suggested by the reviewer. Specifically,
“A major advantage of hot-starting is that it does not require extensive knowledge of the

underlying code to optimize performance. While it may be more efficient to simply re-write the code to eliminate repetitive steps – this is not always feasible especially for academic or poorly documented legacy software. ”

Reviewer #3:
In there manuscript called “Hot-starting software containers for bioinformatics analyses" Pai Zhang and colleagues describing an idea about hot-starting containers and providing some benchmarks with the claim that this could speed up calculations, potentially many, and improving overall performance. This was demonstrated with the well known STAR software for mapping reads.

While the idea on a first glance looks super cool and the graphs promising some major performance difference I have some major concerns and questions.

The authors compare a system where a container is generating the index on the fly, with a system that has a pre-build index in memory. But in reality people do not generate the index on the fly but using pre-build indices that are mounted from external source into the container. It would be interesting to see how much faster this approach is, if the index does not need to be generated but can be mounted as is into the container. Please elaborate on the performance difference between "STAR reads the index into memory" and "mmap reads the memory-container dump".
Pre-generated indices are provided by a lot of different community projects and can be even mounted into containers. Please discuss if the performance gain of your approach is worth the extra steps and the loss in reproducibility and usability.

Our response:
We would like to clarify that we did not generate indices on the fly, and the testing was done with pre-generated indices. We understand and apologize for the loose wording in the original manuscript where we refer to generation of indices. All the experiments were done with pre-generated indices. The initialization step that we are referring to, that we avoid, is the reading of the indices into memory. We have made extensive corrections throughout the abstract, manuscript and figure legends to make this clear.

Specifically, we clarified this point in the following places in the manuscript:
● Abstract (page 2) Findings: “We use an open source tool called Checkpoint and Restore in Userspace (CRIU) to save the state of the containers as a collection of checkpoint files on disk after it has read in the indices.”
● “Our Approach” on page 4: “For STAR, the process of reading in the indices is a slow process and STAR has an option of keeping the indices in memory after they have been generated so that subsequent sequence alignments do not have to repeat the step of reading the indices. We used the CRIU tool to create checkpoints after the indices have been read. “

Assuming hot-starting a container in comparison to using a pre-build index is still faster I would like to see a small discussion about how this compares in price and efficiency. Because with this approach a researcher still needs to transfer and store the memory dump in the cloud - and storage in the cloud is not cheap.

Our response:
The reviewer is absolutely correct. We have not taken into account the charges for long-term

storage onto the cloud for different storage types. We have removed the language referring to cost savings and focus on speed-up which we do directly benchmark.

- Abstract (page 2). “We demonstrate that hot-starting, from containers that have been frozen after the application has already begun execution, can speed up bioinformatics workflows by avoiding repetitive initialization steps.”
- Abstract (page 2). “We demonstrate that hot-starting Docker containers from snapshots taken after repetitive initialization steps are completed, significantly speeds up the execution of the STAR aligner on all experimental platforms …”
- Before “Methods” on page 6. “Hot-starting from pre-initialized containers represents a novel and unexplored approach to speeding up bioinformatics workflows deployed on the cloud or local servers. ”

In the last sentence it was mentioned that these snapshots are more or less not transferable to arbitrary hosts because of the different kernel versions. This is a major drawback of this approach and should be more prominently discussed. How stable is the interface between kernel versions or operating systems? How is reproducibility guaranteed? What can happen if I choose the wrong memory dump?

Our response:
This is true of any containerized application. We rely on Docker to handle the low-level interactions with the kernel and managing reproducibility. We have added more text to the Discussion section to elaborate on this point. Specifically, we added the following sentences on page 6:
“There are several caveats to the hot-start strategy. One is that the CRIU tool is Linux kernel version dependent [18]. Checkpoint files are not portable among hosts where different versions of the Linux kernel are used. However, this is an implicit feature for any container workflow: the reproducibility of components outside the container depend upon a platform-specific implementation of the containerization software. ”

To address your point about how reproducibility, we removed the word “guarantee” in the manuscript. Specifically, on page 3
“Other container technologies such as Singularity containers have also been proposed to enhance mobility and reproducibility of computational science [5, 6]. Thus, containerization enhances the reproducibility of bioinformatics workflows [7-9]. ”

How many times was the experiment repeated to produce Figure 2? A standard deviation is missing in this figure.

Our response:
The experiment results shown in Figure 2 are repeated 5 times. We clarified this point in both the main text and caption for Figure 2.
In addition, we added Additional File 2 that shows the raw results, average, standard deviation and standard errors of alignment time (seconds) for each of the 4 settings (AWS with local disk, AWS EBS, Azure local host, Azure File Storage). We did not add the error bars to Figure 2 because the standard deviations and standard errors for the experiments across the 5 runs are very small compared to the total alignment time, and hence, cannot be shown clearly in the figure.

The authors claim that this approach is usable by other bioinformatics software. I would like

to see at least 2-3 other examples where this speedup is archived. If not please adopt the title and don't make generalize claims.

Our response:
The reviewer's point is well-taken. We have modified the title of the manuscript to be "Hot-starting software containers for STAR aligner" so as to be more specific.

The authors using the term containers, but only mentioned Docker in the manuscript. Is the same technique possible using other container technologies? Singularity or rkt for example?

Our response:
Our manuscript focuses on Docker containers. We added a sentence and two references on Singularity containers in the manuscript in the first paragraph under "Background" to acknowledge that there are other container technologies. Specifically, the following references are added:
5. Kurtzer GM, Sochat V and Bauer MW. Singularity: Scientific containers for mobility of compute. PLoS One. 2017;12 5:e0177459. doi:10.1371/journal.pone.0177459.
6. Sochat VV, Prybol CJ and Kurtzer GM. Enhancing reproducibility in scientific computing: Metrics and registry for Singularity containers. PLoS One. 2017;12 11:e0188511. doi:10.1371/journal.pone.0188511.

Figure 2 has a bad quality this should be improved.

Our response:
Thank you for the suggestion. We have submitted higher resolution figures for both Figure 1 and Figure 2 in this revision.

A citation for the paragraph "Thus, containerization enhances ..." would be nice. There is a lot of literature and big communities in bioinformatics that have studied this topic already.

Our response:
In response to this comment, three references are added to support this statement in the first paragraph under "Background" on page 1. Specifically, the following references are added:

7. Schulz WL, Durant TJ, Siddon AJ and Torres R. Use of application containers and workflows for genomic data analysis. Journal of pathology informatics. 2016;7:53. doi:10.4103/2153-3539.197197.
8. Silver A. Software simplified: Containerization technology takes the hassle out of setting up software and can boost the reproducibility of data-driven research. Nature. 2017;546:173-4.
9. Piccolo SR and Frampton MB. Tools and techniques for computational reproducibility. GigaScience. 2016;5 1:30. doi:10.1186/s13742-016-0135-4.

Close
