# Supplementary material for: Hot-starting software containers for STAR aligner
Source: Gigascience. 2018 Jul 31;7(8):giy092. doi: 10.1093/gigascience/giy092 (PMC6131214; doi:10.1093/gigascience/giy092)
Supplement: Response_to_Reviewer_Comments_Revision1.pdf [file giy092_response_to_reviewer_comments_revision1.pdf]

Author's Response To Reviewer Comments

Close

Thank you for the opportunity to publish in Gigascience. We added the following reference to the manuscript as instructed.

[22] Zhang, P; Hung, L, H; Lloyd, W; Yeung, K, Y (2018): Supporting data for"Hot-starting software containers for STAR aligner" GigaScience Database. http://dx.doi.org/10.5524/100468

Close
