# Supplementary material for: Hot-starting software containers for STAR aligner
Source: Gigascience. 2018 Jul 31;7(8):giy092. doi: 10.1093/gigascience/giy092 (PMC6131214; doi:10.1093/gigascience/giy092)
Supplement: Reviewer_1_Report_(Original_Submission) -- Mark Fernandes — 1/15/2018 Reviewed [file giy092_reviewer_1_report_(original_submission).pdf]

Reviewer Report

Title: Hot-starting software containers for STAR aligner

Version: Original Submission **Date:** 1/15/2018

Reviewer name: Mark Fernandes

Reviewer Comments to Author:

This paper introduces the concept that Cloud computing and Containers offer new routes to pipeline optimisation.I like the use of multiple Cloud providers which highlights issues with optimisation for different network filesystems.It would have been interesting to have a comparison with a typical HPC approach e.g. saving the pre-computedindices to a parallel filesystem such as Lustre to highlight the optimisation wrt retaining indices in memory.With respect to HPC CRIU may also have some utility there e.g. with Singularity containers which are often accepted byCluster admins compared to Docker.I visited the web resources for the softare and found them fairly comprehensive and active.

Level of Interest

Please indicate how interesting you found the manuscript: An article of importance in its field

Quality of Written English

Please indicate the quality of language in the manuscript: Acceptable

Declaration of Competing Interests

Please complete a declaration of competing interests, considering the following questions:

- Have you in the past five years received reimbursements, fees, funding, or salary from an organisation that may in any way gain or lose financially from the publication of this manuscript, either now or in the future?
- Do you hold any stocks or shares in an organisation that may in any way gain or lose financially from the publication of this manuscript, either now or in the future?
- Do you hold or are you currently applying for any patents relating to the content of the manuscript?
- Have you received reimbursements, fees, funding, or salary from an organization that holds or has applied for patents relating to the content of the manuscript?
- Do you have any other financial competing interests?
- Do you have any non-financial competing interests in relation to this paper?

If you can answer no to all of the above, write 'I declare that I have no competing interests' below. If your reply is yes to any, please give details below.

declare that I have no competing interest

I agree to the open peer review policy of the journal. I understand that my name will be included on my report to the authors and, if the manuscript is accepted for publication, my named report including any attachments I upload will be posted on the website along with the authors' responses. I agree for my report to be made available under an Open Access Creative Commons CC-BY license (http://creativecommons.org/licenses/by/4.0/). I understand that any comments which I do not wish to be included in my named report can be included as confidential comments to the editors, which will not be published.

I agree to the open peer review policy of the journal

o further support our reviewers, we have joined with Publons, where you can gain additional credit to further highlight your hard work (see: https://publons.com/journal/530/gigascience). On publication of this paper, your review will be automatically added to Publons, you can then choose whether or not to claim your Publons credit. I understand this statement. Yes
