# Supplementary material for: Hot-starting software containers for STAR aligner
Source: Gigascience. 2018 Jul 31;7(8):giy092. doi: 10.1093/gigascience/giy092 (PMC6131214; doi:10.1093/gigascience/giy092)
Supplement: Reviewer_2_Report_(Original_Submission) -- Francois Moreews — 1/23/2018 Reviewed [file giy092_reviewer_2_report_(original_submission).pdf]

Reviewer Report

Title: Hot-starting software containers for STAR aligner

Version: Original Submission **Date:** 1/23/2018

Reviewer name: Francois Moreews

Reviewer Comments to Author:

The authors propose a new technique to optimise the cost and execution time of data analysis using containers in batch processing mode.This technique relies on an existing tool named CRIU (Checkpoint and Restore in Userspace)that allows to "freeze" the state of a linux process and acts as a "snapshot" including RAM state.CRIU persists a state of a running application to a hard drive as a collection of files.The innovation described in the paper consists in the application of this checkpoint technique to data processing tools executed in Docker containers.The detailed benchmark is based on the STAR aligner, a sequence alignment tool used for high-throughput RNA-seq data.In the use case described in the paper, the container executing the software is frozen after the reference index creation, a costly initial step.Then, the "snapshot" container can be reused in a loop, iterating the whole data collection, but without the cost of the index creation.Then , the benchmark shows that the method reduces the aligner execution time.Even if CRIU is already available as an experimental feature in Docker, Openvz and LXC, the described work brings something new. Based on our current knowledge and referenced publications, the idea of using "frozen containers" to reduce the time and cost of execution does not seem to have ever been published before.The proposition of using these "Hot-starting software containers" to improve the performance of bioinformatics data analysis looks promising especially combined with data parallelisation.It can have a strong impact on cost and execution time of high throughput data analysis using containers like Docker or LXC, especially on a commercial cloud.We suggest to precise that the described method is especially interesting in the context of "heterogeneous" and "legacy" software integration which is not covered in the paper.Indeed, a classical approach to optimise STAR is its direct code modification. A coded new feature that allows to reuse and existing persisted index can produce a similar optimisation.The authors may explain that this naive solution is not always possible because bioinformaticians are reusing a lot of tools considered as "black boxes" and sometimes the tools are simply not maintained any more.A bioinformatics workflow generally embeds external legacy software building blocks developed by multiple authors and the workflow developer is often not the author of the building blocks.In these cases, common in bioinformatics, the "Hot-starting software containers" optimisation technique can be very useful.I suggest also to put emphasis on the fact that the technique is as well important for speed-up workflows, not only for cost. Moreover, containers can be used on local PC and not only on clouds.

Level of Interest

Please indicate how interesting you found the manuscript: An article of importance in its field

Quality of Written English

Please indicate the quality of language in the manuscript: Acceptable

Declaration of Competing Interests

Please complete a declaration of competing interests, considering the following questions:

- Have you in the past five years received reimbursements, fees, funding, or salary from an organisation that may in any way gain or lose financially from the publication of this manuscript, either now or in the future?
- Do you hold any stocks or shares in an organisation that may in any way gain or lose financially from the publication of this manuscript, either now or in the future?
- Do you hold or are you currently applying for any patents relating to the content of the manuscript?
- Have you received reimbursements, fees, funding, or salary from an organization that holds or has applied for patents relating to the content of the manuscript?
- Do you have any other financial competing interests?
- Do you have any non-financial competing interests in relation to this paper?

If you can answer no to all of the above, write 'I declare that I have no competing interests' below. If your reply is yes to any, please give details below.

I declare that I have no competing interests

I agree to the open peer review policy of the journal. I understand that my name will be included on my report to the authors and, if the manuscript is accepted for publication, my named report including any attachments I upload will be posted on the website along with the authors' responses. I agree for my report to be made available under an Open Access Creative Commons CC-BY license (http://creativecommons.org/licenses/by/4.0/). I understand that any comments which I do not wish to be included in my named report can be included as confidential comments to the editors, which will not be published.

I agree to the open peer review policy of the journal

To further support our reviewers, we have joined with Publons, where you can gain additional credit to further highlight your hard work (see: https://publons.com/journal/530/gigascience). On publication of this paper, your review will be automatically added to Publons, you can then choose whether or not to claim your Publons credit. I understand this statement. Yes
