# Supplementary material for: Hot-starting software containers for STAR aligner
Source: Gigascience. 2018 Jul 31;7(8):giy092. doi: 10.1093/gigascience/giy092 (PMC6131214; doi:10.1093/gigascience/giy092)
Supplement: Reviewer_3_Report_(Original_Submission) -- Björn Grüning — 2/12/2018 Reviewed [file giy092_reviewer_3_report_(original_submission).pdf]

Reviewer Report

Title: **Hot-starting software containers for STAR aligner**

Version: **Original Submission** **Date:** 2/12/2018

Reviewer name: **Björn Grüning**

Reviewer Comments to Author:

In there manuscript called „Hot-starting software containers for bioinformatics analyses" Pai Zhang and colleagues describing an idea about hot-starting containers and providing some benchmarks with the claim that this could speed up calculations, potentially many, and improving overall performance. This was demonstrated with the well known STAR software for mapping reads.While the idea on a first glance looks super cool and the graphs promising some major performance difference I have some major concerns and questions.* The authors compare a system where a container is generating the index on the fly, with a system that has a pre-build index in memory. But in reality people do not generate the index on the fly but using pre-build indices that are mounted from external source into the container. It would be interesting to see how much faster this approach is, if the index does not need to be generated but can be mounted as is into the container. Please elaborate on the performance difference between "STAR reads the index into memory" and "mmap reads the memory-container dump".* Assuming hot-starting a container in comparison to using a pre-build index is still faster I would like to see a small discussion about how this compares in price and efficiency. Because with this approach a researcher still needs to transfer and store the memory dump in the cloud - and storage in the cloud is not cheap.* In the last sentence it was mentioned that these snapshots are more or less not transferable to arbitrary hosts because of the different kernel versions. This is a major drawback of this approach and should be more prominently discussed. How stable is the interface between kernel versions or operating systems? How is reproducibility guaranteed? What can happen if I choose the wrong memory dump?* Pre-generated indices are provided by a lot of different community projects and can be even mounted into containers. Please discuss if the performance gain of your approach is worth the extra steps and the loss in reproducibility and usability.* How many times was the experiment repeated to produce Figure 2? A standard deviation is missing in this figure.* The authors claim that this approach is usable by other bioinformatics software. I would like to see at least 2-3 other examples where this speedup is archived. If not please adopt the title and don't make generalize claims.Minor things:* The authors using the term containers, but only mentioned Docker in the manuscript. Is the same technique possible using other container technologies? Singularity or rkt for example?* Figure 2 has a bad quality this should be improved.* A citation for the paragraph "Thus, containerization enhances ..." would be nice. There is a lot of literature and big communities in bioinformatics that have studied this topic already.With kind regards,Bjoern Gruening

Level of Interest

Please indicate how interesting you found the manuscript: An article of limited interest

Quality of Written English

Please indicate the quality of language in the manuscript: Acceptable

Declaration of Competing Interests

Please complete a declaration of competing interests, considering the following questions:

- Have you in the past five years received reimbursements, fees, funding, or salary from an organisation that may in any way gain or lose financially from the publication of this manuscript, either now or in the future?
- Do you hold any stocks or shares in an organisation that may in any way gain or lose financially from the publication of this manuscript, either now or in the future?
- Do you hold or are you currently applying for any patents relating to the content of the manuscript?
- Have you received reimbursements, fees, funding, or salary from an organization that holds or has applied for patents relating to the content of the manuscript?
- Do you have any other financial competing interests?
- Do you have any non-financial competing interests in relation to this paper?

If you can answer no to all of the above, write 'I declare that I have no competing interests' below. If your reply is yes to any, please give details below.

I declare that I have no competing interests.

I agree to the open peer review policy of the journal. I understand that my name will be included on my report to the authors and, if the manuscript is accepted for publication, my named report including any attachments I upload will be posted on the website along with the authors' responses. I agree for my report to be made available under an Open Access Creative Commons CC-BY license (http://creativecommons.org/licenses/by/4.0/). I understand that any comments which I do not wish to be included in my named report can be included as confidential comments to the editors, which will not be published.

I agree to the open peer review policy of the journal

To further support our reviewers, we have joined with Publons, where you can gain additional credit to further highlight your hard work (see: https://publons.com/journal/530/gigascience). On publication of this paper, your review will be automatically added to Publons, you can then choose whether or not to claim your Publons credit. I understand this statement. Yes
