# Supplementary material for: Hot-starting software containers for STAR aligner
Source: Gigascience. 2018 Jul 31;7(8):giy092. doi: 10.1093/gigascience/giy092 (PMC6131214; doi:10.1093/gigascience/giy092)
Supplement: Reviewer_3_Report_(Revision_1) -- Björn Grüning — 5/13/2018 Reviewed [file giy092_reviewer_3_report_(revision_1).pdf]

Reviewer Report

Title: **Hot-starting software containers for STAR aligner**

Version: **Revision 1** **Date:** 5/13/2018

Reviewer name: **Björn Grüning**

Reviewer Comments to Author:

Thanks for addressing my comments. I have a few more comments to your adjustments.

>> In the last sentence it was mentioned that these snapshots are more or less not transferable to arbitrary hosts because of the different kernel versions. This is a major drawback of this approach and should be more prominently discussed. How stable is the interface between kernel versions or operating systems? How is reproducibility guaranteed? What can happen if I choose the wrong memory dump?

> Our response:
This is true of any containerized application. We rely on Docker to handle the low-level interactions with the kernel and managing reproducibility. We have added more text to the Discussion section to elaborate on this point. Specifically, we added the following sentences on page 6:

This is not true. Docker images are following the OSI container specification and they can be exchanged and are not bound
to any specific kernel version. The limitation which kicks in here, is the limitation of the memory dump and this is new
and restricts the usefulness of the approach dramatically. For example the cluster which needs to run the workflow
needs to have the exact same kernel version as far as I understand.

> "There are several caveats to the hot-start strategy. One is that the CRIU tool is Linux kernel version dependent [18]. Checkpoint files are not portable among hosts where different versions of the Linux kernel are used. However, this is an implicit feature for any container workflow: the reproducibility of components outside the container depend upon a platform-specific implementation of the containerization software. "

All caveats should be discussed not only one.
I disagree with the "implicit feature", see above. We are discussing here the non-portable memory dump and not that Docker containers
are dependent on specific kernel features.
Also the term "implicit feature" is confusing here as this is a limitation.

Methods

Are the methods appropriate to the aims of the study, are they well described, and are necessary controls included? Choose an item.

Conclusions

Are the conclusions adequately supported by the data shown? Choose an item.

Reporting Standards

Does the manuscript adhere to the journal's guidelines on minimum standards of reporting? Choose an item.

Choose an item.

Statistics

Are you able to assess all statistics in the manuscript, including the appropriateness of statistical tests used? Choose an item.

Quality of Written English

Please indicate the quality of language in the manuscript: Choose an item.

Declaration of Competing Interests

Please complete a declaration of competing interests, considering the following questions:

- Have you in the past five years received reimbursements, fees, funding, or salary from an organisation that may in any way gain or lose financially from the publication of this manuscript, either now or in the future?
- Do you hold any stocks or shares in an organisation that may in any way gain or lose financially from the publication of this manuscript, either now or in the future?
- Do you hold or are you currently applying for any patents relating to the content of the manuscript?
- Have you received reimbursements, fees, funding, or salary from an organization that holds or has applied for patents relating to the content of the manuscript?
- Do you have any other financial competing interests?
- Do you have any non-financial competing interests in relation to this paper?

If you can answer no to all of the above, write 'I declare that I have no competing interests' below. If your reply is yes to any, please give details below.

I declare that I have no competing interests

I agree to the open peer review policy of the journal. I understand that my name will be included on my report to the authors and, if the manuscript is accepted for publication, my named report including any attachments I upload will be posted on the website along with the authors' responses. I agree for my report to be made available under an Open Access Creative Commons CC-BY license (http://creativecommons.org/licenses/by/4.0/). I understand that any comments which I do not wish to

be included in my named report can be included as confidential comments to the editors, which will not be published.

I agree to the open peer review policy of the journal

To further support our reviewers, we have joined with Publons, where you can gain additional credit to further highlight your hard work (see: https://publons.com/journal/530/gigascience). On publication of this paper, your review will be automatically added to Publons, you can then choose whether or not to claim your Publons credit. I understand this statement.

Yes Choose an item.
